# Action-effect related motor adaptation in interactions with everyday devices

**DOI:** 10.1038/s41598-018-25161-w

**Published:** 2018-04-26

**Authors:** János Horváth, Botond Bíró, Bence Neszmélyi

**Affiliations:** 1 Institute of Cognitive Neuroscience and Psychology, Research Centre for Natural Sciences, Hungarian Academy of Sciences, Budapest, Hungary; 2 Budapest University of Technology and Economics, Budapest, Hungary

## Abstract

Human action planning relies on integrated representations of motor acts and the associated consequences, which implies that changing the set of effects associated to a motor act might directly influence action planning and control. The present study investigated the hypothesis that action-effect manipulations also affected the motor components of the actions even when only a single action option was available. Participants performed simple everyday actions (pinched a plastic sheet, pressed a button, tapped on a table) in two conditions. In the motor-auditory condition actions resulted in the presentation of a tone, whereas no tones were presented in the motor condition. The applied force was softer in the motor-auditory than in the motor condition for all three types of actions. The temporal characteristics of force application showed that action-effect related motor adaptation occurred during action planning, but possibly also during action execution. This demonstrates that even in simple, well-defined interactions with everyday devices we take all (even seemingly task-irrelevant) action-effects into account during action planning, which affects the motor component of the action. The results also imply that in experiments manipulating contingent action effects, one cannot rely on the assumption that the motor part of the action is invariant between conditions.

## Introduction

Most of our actions have multiple consequences. Some of these effects are the reasons why we perform the actions in the first place, that is, these are the goals of the actions, whereas others are merely side-effects. When pressing the button of the doorbell, our intention is to initiate a sound that signals the residents of the house that we have arrived. Besides initiating the sound, pressing the button also results in proprioceptive, tactile and visual stimulation. As long as pressing the button results in the doorbell sound, these other consequences are irrelevant side-effects. If the action fails (no sound is heard), however, side-effects may become important in correcting the failure: we might think that we did not press the button properly, and may attempt some more presses: maybe this time looking at the button to ensure that we really press its moving element, maybe we press with more force to make sure that the button reaches its end position, and one may also listen to whether the button clicks or not.

The predictable sensory consequences of our actions play an important role in action planning, and action planning influences sensory preparation for action effects (see e.g., Rizzolatti, *et al*.^1^; Baldauf & Deubel^2^), which suggests that these effects form an integral part of our action representations^3^. Although the integration of sensory consequences into the action representation is automatic^4,5^, we also have considerable freedom to structure such representations in accord with our intentions^6^. For example, the same actions can be represented with proximal or with distal action-effects, which result in different interference patterns in action planning^7,8^, and it also enables efficient tool use^9^. If the encoding of action consequences plays such an important role in action planning, then it seems plausible that physically changing the sensory consequences of an action (e.g. removing one of the action effects, especially the one preferentially used to encode the action) also leads to marked changes in action planning. One may even speculate that such adjustments lead to changes in the *motor part* of the action. The goal of the present study was to investigate whether differences in associated effects were also reflected in the motor components of the actions.

Most studies investigating the influence of action-effect representations on action planning administered paradigms with explicit mappings between several *categories* of actions and corresponding effects (e.g. button presses categorized as soft or strong elicited a soft or a loud sound^10^; or buttons pressed for short or long intervals elicited a short or a long tone^11^). Such *response-effect compatibility paradigms* demonstrated that actions consistently eliciting effects with incompatible features (e.g. soft button presses eliciting the loud tone^10^; or short-duration button presses eliciting the long tone^11^) were initiated more slowly (after an imperative cue was presented) than those consistently eliciting effects with compatible ones. Various experiments^12,13^ showed that in the presence of multiple action options, the trajectories of manual reaching movements – while still reaching their goals – may reflect bias towards not-chosen action options, which may allow insights into the acivation patterns of action representations within the given context (e.g. Wirth *et al*.^14^).

In contrast with response-effect compatibility paradigms, the present study utilized a *single* action category, that is, there were no alternative response options; and measured the motor parameters of the actions within that single action category. The notion that motor parameters within a single category of actions may change even in the absence of an alternative action category, as a function of the elicited effects, is supported by a recent study by Neszmélyi, & Horváth^15^, in which actions with and without auditory consequences (in separate experimental blocks) were compared. It was found that participants applied less force when actions elicited a sound (*action-effect related force adaptation*). An important implication of this result is that action-effect manipulations may not only influence the sensory input induced by the action, but the motor output as well, which challenges the validity of motor correction methods often applied in electroencephalographic, magnetoencephalographic, or brain imaging studies on the processing of self-induced tones. In such studies, it is often assumed that the motor part of the action is the same irrespectively of the elicited effects, and sensory processing activity related to the self-induced action-effect is estimated by subtracting the processing activity measured when the actions are performed without bringing the given effect about^16^.

The action used in the Neszmélyi and Horváth^15^ study was, however, somewhat peculiar. Participants were pinching a small plastic sheet (i.e., they held it between the thumb and the index finger), and were instructed to apply brief force impulses in the context of a time interval production task. An action was registered when pinch-force exceeded a pre-set threshold level, which, in one condition, resulted in the immediate presentation of a tone. Although this pattern of force application is similar for many common types of interactions (e.g. pressing a button), this particular action-device combination is seldom used in everyday life. Whereas more common devices often produce a distinct mechanical (tactile) transient when the action is successfully executed (e.g., after a button moves, it hits its end-position), the tactile sensations produced during pinching a plastic sheet are probably much less distinct. That is, pinching may not have the tactile effects that define successful interactions with most everyday devices, thus may not allow participants to encode pinching in “tactile terms” as, for example, button presses may do. Therefore, one may argue that the action-effect related force adaptation reported by Neszmélyi and Horváth^15^ was specific to pinching, and may not reflect a general phenomenon. The goals of the three experiments of the present study were to replicate the original results (Pinch Experiment), and to test whether action-effect related adaptation could also be observed for more frequently used types of interactions: tapping (Tap Experiment) and button pressing (Button Experiment). In all three experiments participants repeated the given action in an even tempo (one action every three seconds) in two separate conditions: in the *motor-auditory* condition, each action resulted in the elicitation of a tone, whereas in the *motor* condition actions did not elicit a tone. The applied force was continuously measured by force sensitive resistors (FSRs).

## Results

**Figure 1:**
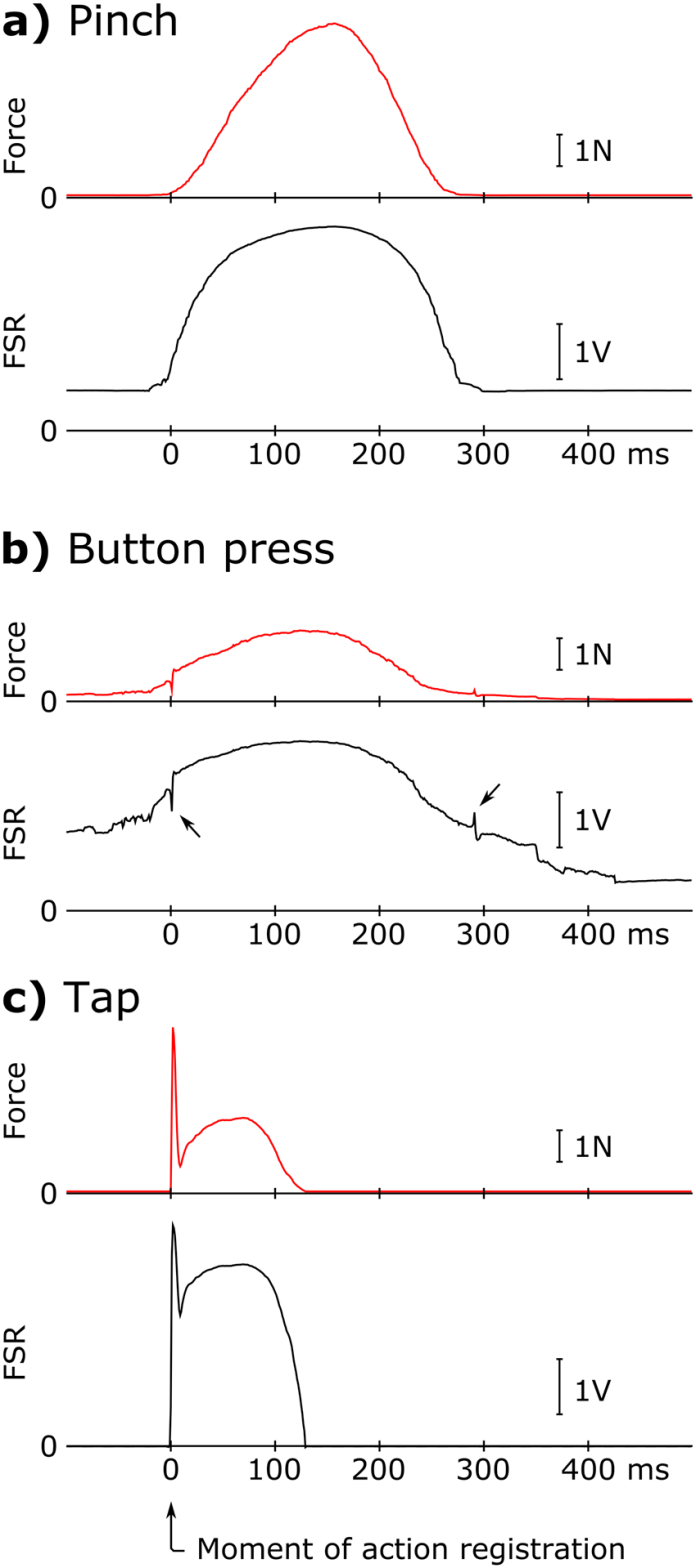
Representative FSR signals, and the corresponding force signals for single actions in the Pinch (**a**), Button press (**b**) and Tap (**c**) experiments. The actions were registered at 0 ms (corresponding to the moment when the applied force exceeded pre-set thresholds in the Pinch (**a**) and Tap (**c**) experiments, and the moment the button was actuated in the Button experiment (**b**). In panel **b**), arrows point to button-displacement related transients in the FSR signal.

Participants complied with the instructions. Between-action intervals (Table 1) did not significantly differ between conditions in any of the three experiments. The interactions with the different devices resulted in different FSR and force signal time courses (Fig. 1).

**Table 1:** Group-mean between-action intervals with standard deviations in the two conditions of the three experiments.

Experiment	Between-action interval (ms)		Comparison
	Motor condition	Motor-Auditory condition	
Pinch	3167 (926)	2803 (970)	*t*(15) = 1.736, *p* = 0.103, *d* = 0.434
Button	2903 (990)	2864 (1307)	*t*(15) = 0.327, *p* = 0.756, *d* = 0.082
Tap	2900 (469)	3206 (807)	*t*(15) = −1.502, *p* = 0.154, *d* = 0.376

Comparisons are paired, two-tailed Student’s *t* tests, with *d* effect size^25^. Note that the elimination of actions preceding or following another action within 1 s – a measure taken to eliminate erroneously registered actions, see Methods – affected only 1 ± 3% (mean and standard deviation, range: 0–18%) of the registered actions in each participant and block.

### Pinch Experiment

In the Pinch Experiment the FSR and corresponding force signal (Fig. 1a) showed a reversed U-shaped curve, in some cases with a sustained period of maximal force exertion. Since participants held the device between their fingers, a (mostly) constant, below-threshold signal could be observed before and after the pinches. Each action was characterized by the maximal force peak, which could be identified for all the actions in the motor, and for 99.7 ± 0.4% (minimum 99.0%) of the actions in the motor-auditory conditions (no significant difference, *t*[15] = 1.464, *p* = 0.164). The force application pattern was characterized for each participant by the median of peak forces, and the mean latency of the identified peaks. The applied force peaked later (Table 2), and was significantly higher (Table 3) in the motor than in the motor-auditory condition (see also Supplementary Fig. S1).

**Table 2:** Group-mean FSR/force signal peak latencies with standard deviations in the three experiments.

Peak	Peak latency (ms)		Comparison
	Motor condition	Motor-Auditory condition	
Pinch	289 (132)	154 (84)	*t*(15) = 6.163, *p* < 0.001, *d* = 1.154
Button	77 (22)	66 (10)	*t*(15) = 2.502, *p* = 0.024, *d* = 0.626
Tap 1^st^	4.6 (2.7)	6.1 (4.1)	*t*(15) = −3.837, *p* = 0.002, *d* = 0.959
Tap 2^nd^	57 (27)	44 (14)	*t*(15) = 2.517, *p* = 0.024, *d* = 0.629

Comparisons are paired, two-tailed Student’s t-tests, with *d* effect size^25^. Note the opposite sign of the effect for the first peak latency in the Tap Experiment (in comparison to the other three peaks).

**Table 3:** Group-median peak FSR signal- and the corresponding force amplitudes, with inter-quartile ranges in the motor and motor-auditory conditions of the three experiments.

Peak	FSR signal amplitude (V)		Force amplitude (N)		Between-condition force comparison
	Motor condition	Motor-Auditory condition	Motor condition	Motor-Auditory condition	
Pinch	3.83 (0.81)	2.71 (1.48)	6.71 (0.09)	1.33 (0.22)	*T* = 0, *p* < 0.001, *r* = 1.000
Button	2.53 (0.33)	2.37 (0.18)	1.03 (0.04)	0.82 (0.04)	*T* = 22, *p* = 0.016, *r* = 0.676
Tap 1^st^	3.15 (0.81)	2.30 (0.97)	2.05 (0.15)	0.80 (0.18)	*T = *4, *p* < 0.001, *r* = 0.941
Tap 2^nd^	2.88 (1.24)	1.51 (1.41)	1.53 (0.25)	0.33 (0.29)	*T* = 0, *p* < 0.001, *r* = 1.000

Comparisons are Wilcoxon signed rank tests, the *r* effect size is the matched-pairs rank biserial correlation coefficient^26,27^.

### Button Experiment

For button-presses, the FSR and corresponding force signals were largely similar to those for pinches: they showed a reversed U-shaped curve with a single peak (Fig. 1b). There were two conspicuous differences: First, button displacements could be observed as sharp transients superimposed on the reversed U-shaped signal curve (indicated by arrows in Fig. 1b). These transients reflect the mechanical operation of the button: when the applied force reaches the mechanical threshold of the button, the signal drops as the inward displacement starts, then quickly rises as it is stopped at the end position. Then, in a pressed state, as the applied force decreases, the button pops out, “hitting” the finger, which results in a transient increase, followed by a decrease in force. Second, before pressing the button, participants applied above-baseline force to the button; in some cases even close-to-threshold force was maintained before the button press (observable in Fig. 1b, when comparing the FSR signal before the button press and after the button release). This pattern of force application may reflect the familiarity with the operation of a button, as maintaining a close-to-threshold force allows faster button actuation when the decision to press the button is made. As in the Pinch Experiment, each action was characterized by the force peak of the U-shaped curve. To exclude force peaks of button-displacement-related transients from the peak search, the peak search interval started 20 ms after the actuation of the button (i.e. 20 ms after the action was registered). A force peak could be identified in the search interval for 99.7 ± 0.5% (min. 98.6%) of the actions in the motor, and for 99.8 ± 0.4% (min. 98.9%) of the actions in the motor-auditory conditions (no significant difference, *t*[15] = 0.760, *p* = 0.459). As in the Pinch Experiment, the applied force peaked later (Table 2), and the peak was significantly higher (Table 3) in the motor than in the motor-auditory condition (see also Supplementary Fig. S2).

### Tap Experiment

For taps, the FSR and force signals do not capture the full time course of the tapping movement, because the FSR shows a signal change only when contact is made, that is, when the participant’s finger touches the FSR. In the typical response pattern, as the finger hits the surface, the signal rises quickly and reaches its peak ca. within the first 20 ms, reflecting the deceleration of the finger (Fig. 1c). However, at this time point the interaction is not over yet, in most cases the participant’s force application continues, which produces a second, longer lasting peak. This second peak was not present for all actions: for some actions, especially in the motor-auditory condition, no second peak could be identified (see below).

For the first peak, a force peak could be identified for 99.3 ± 1.7% (min. 93.5%) of the actions in the motor, and for 98.0 ± 4.3% (min. 87.5%) of the actions in the motor-auditory conditions (no significant difference, *t*[15] = 1.058, *p* = 0.307). For the second peak, a peak could be found for 99.6 ± 0.8% (min. 96.9%) of the actions in the motor, and for 95.2 ± 7.3% (min. 74.0%) of the actions in the motor-auditory condition (*t*[15] = 2.392, *p* = 0.030). That is, a significantly lower number of peaks could be identified in the second search interval in the motor-auditory condition.

Peak force amplitudes were higher in the motor than in the motor-auditory condition for both peaks (Table 3). In contrast with the other peaks (in all three experiments), the latency of the first peak was earlier in the motor than in the motor-auditory condition (see also Supplementary Figs S3 and S4). This result indicates that the initial momentum of the tapping finger is larger, which leads to a sharper impact-related transient in the motor condition.

### Within-block peak force and latency correlations

Although in the above analyses force peak amplitudes and latencies were analyzed separately, the visual inspection of the data (Supplementary Figs S1, S2, S3, and S4) suggested that force peak amplitude and latency were correlated in most participants. To explore this impression, Spearman rank-correlations were calculated for each participant in each condition, which were submitted to Student’s *t* tests against zero. The results (Table 4) confirmed that higher peak amplitudes were indeed reached later in the Pinch and Button Experiments. In the motor-auditory condition of the Tap Experiment, higher amplitude first peaks occurred earlier, whereas higher amplitude second peaks occurred later. Note that in the Tap Experiment, the sampling interval (one sample per ms) was relatively low with respect to the latencies of the first peaks (see Table 2), which lead to numerous ties in the calculation of the correlations.

**Table 4:** Group-mean Spearman rank correlation coefficients between individual force peak amplitudes and latencies (with standard deviations in parentheses), as well as their one-sample two-tailed Student’s t-test comparisons against zero with *d* effect size^25^ in the motor and motor-auditory conditions of the three experiments.

Peak	Correlation	
	Motor condition	Motor-Auditory condition
Pinch	0.31 (0.25) *t*(15) = 5.068, *p* < 0.001, *d* = 1.267	0.49 (0.21) *t*(15) = 9.089, *p* < 0.001, *d* = 2.272
Button	0.37 (0.15) *t*(15) = 9.635, *p* < 0.001, *d* = 2.409	0.32 (0.15) *t*(15) = 8.617, *p* < 0.001, *d* = 2.154
Tap 1^st^	−0.12 (0.33) *t*(15) = −1.491, *p* = 0.157, *d* = −0.373	−0.21 (0.27) *t*(15) = −3.167, *p* = 0.006, *d* = −0.792
Tap 2^nd^	0.09 (0.30) *t*(15) = 1.197, *p* = 0.250, *d* = 0.299	0.22 (0.31) *t*(15) = 2.901, *p* = 0.011, *d* = 0.725

## Discussion

The present study tested whether motor parameters of various, simple actions with and without auditory action-effects differed. For pinch impulses applied on a thin plastic sheet, we replicated the finding that participants exerted more force when the auditory effect was consistently absent^15^. Importantly, the same was found for tapping on a table and pressing a button, which are interactions more often utilized in everyday life, as well as in experimental paradigms in psychology and neuroscience. This suggests that action-effect dependent force application differences are not specific to pinching, but reflect a general phenomenon that occurs during various interactions.

Although most of the between-condition differences certainly reflect differences in action planning, in the Pinch Experiment the difference may also receive a contribution from on-line action control. For pinches - because peak latencies are commensurate or longer than typical simple reaction times (Table 2) - it seems possible that participants respond to sound onset (occurring right after the force threshold is exceeded) by initiating a pinch-release (that is, they adjust the overall force-application on the fly), which results in lower and also earlier force peaks than in the motor condition. On the other hand, it is unrealistic that such an adjustment would take place for the initial tapping movement, for which differences in the first force peak occurred typically just as sound presentation started. That is, peak force differences for the first peak in the Tap Experiment certainly reflect differences in action planning. Similarly, given that the latencies of second tap and the button press force peaks were well below the latency of typical reaction times (Table 2, and Supplementary Figs S2 and S4), it seems unlikely that between-condition peak force differences in these experiments received substantial contribution from on-the fly control processes.

In contrast with response-effect compatibility paradigms^10^, the present paradigm offered no alternative action options, therefore the action-effect dependent motor parameter differences cannot reflect concurrent activations of alternative action categories. Although the present study does not allow drawing conclusions on the cause of between-condition differences, a number of hypotheses can be put forward. As speculated previously, there are several factors that may contribute to the observed differences. The following lines of thought suggest that motor parameter differences reflect the optimization of the action (*action-optimization-hypothesis*^15^).

The objectives of such an action optimization can be manifold. On their own, actions in the present experiments resulted in proprioceptive, tactile, visual (because participants may look at their hands), and even auditory feedback (because tapping impact, as well as button clicking is often audible)^17^. One may argue that in such setups, the optimization goal is to maintain a constant level of *overall* feedback intensity, and thus adding a tone compels participants to attenuate the other - motor-parameter dependent - forms of feedback. In the present paradigm, they may lower the contribution of the tactile feedback by reducing the applied force. Indeed, this has been suggested by Kunde and colleagues^18^, who found that peak forces were lower when actions elicited loud sounds than when they elicited soft sounds in an experiment mapping soft and strong button-presses to soft or loud tones.

Although the level of applied force was irrelevant as long as it exceeded the pre-set threshold during the interactions, force cannot be considered as a task-irrelevant action aspect in the present experiments (for an interaction rule in which force is largely task-irrelevant, see Kunde and colleagues)^18^. Thus a different hypothesis regarding the objective of the optimization, which also fits the results, is that participants experience uncertainty regarding whether the applied force would exceed the threshold for a given action instance. Thus participants may try to ascertain that the interactions are successful while keeping the level of effort reasonable. That is, the objective is to maintain a balance between conserving energy (i.e. reducing effort) while keeping the rate of success acceptably high^15^. In this line of thought, the fact that adding a fully contingent tone (i.e., a tone that was presented if, and only if a successful interaction took place) affected motor parameters at all would indicate that in the absence of the tone, the available feedback was not sufficient for the optimization of the effort/success ratio (even for tapping and pressing a button, both of which feature well-identifiable interaction-related tactile transients). That is, the tone might be more distinctive, more well-defined than the other (i.e., tactile and proprioceptive) sources of sensory feedback and thus provide a more reliable signal for action success.

The notion that action consequences may not be “equal” in the present paradigm fits the idea that action effects may not have equal weight in action representations^3–9^. One may speculate that the tone may allow the formation of an action-representation that is more advantageous than the action representation which only included the other (tactile, etc.) action effects. The tone may serve as a central, clear-cut goal for the actions, and thus representing the action in terms of its auditory instead of the other sensory consequences may thus enhance action planning and action-control processes. That is, the action-effect related motor optimization may reflect a difference in action goals or intentions of the participants: in the motor conditions, participants may actually encode the actions as “pinching”, “pressing”, or “tapping”, whereas in the motor-auditory conditions they may actually encode them as “eliciting tones”.

Finally, it can be noted that the speculations summarized above are formulated in terms of *force*-optimization. Since the success of an action depended on exceeding a force threshold, this seems plausible. Because force peak amplitude and latency were positively correlated for the non-impact-related force peaks in the present experiments, one might also propose, however, that the tone-related motor adjustments may not have the primary goal to reduce force, but to reduce the temporal separation between tone onset (occurring close to force onset) and the force peak, which also corresponds to the point of maximal tactile reafference. That is, it is seems possible that the objective is to reduce temporal separation between the different action effects. The notion that the integration of temporally distant action effects into a joint action representation involves binding the events to a common time-point between the two events has been suggested in numerous studies^19,20^. In the present context, it seems plausible that given the opportunity, participants may facilitate the binding of sound onset and tactile feedback by performing the action so that it brings the two events temporally closer together.

The present study extends the range of paradigms that may allow insights into the cognitive determinants of action planning through the measurement of motor parameters. In the context of the present study, more forceful actions may signal that the actor has less confidence that a given action will result in a successful interaction, that is, changes in the distance from the optimum may reflect changes in the level of confidence. Whereas in the present study the set of action-effects were manipulated, it may be possible to reverse the direction of inference, and attribute differences in applied force (for example, between trials) without physical action-effect differences to differences in the cognitive representations of the actions (e.g. intentions or goals of the actor). This idea has been suggested before in the context of object manipulation (for a recent summary, see Rosenbaum *et al*.^21^).

In summary, these robust results demonstrate that action planning and possibly action control activities differ as a function of action-effects even for conventional interaction devices. Adding an auditory action-effect resulted in less forceful actions when pressing a button, or tapping on a table, similarly to pinching. This suggests that task-irrelevant action-effects are not ignored in such a setting, but are actively utilized by the participants to maintain the interaction with the response device over the course of the experiment. Furthermore, the results imply that in experiments manipulating contingent action effects, one cannot rely on the assumption that the motor part of the action is invariant between conditions.

## Methods

### Participants

Sixteen young adults (ten women, age 20–28 years, fourteen right handed) participated in the Tap; sixteen (eleven women; age 19–30 years, fifteen right handed) in the Pinch; and sixteen (nine women; age 19–27 years, thirteen right handed) in the Button Experiment, either for monetary compensation or for course credit. None of the participants participated in more than one of the experiments. In all three experiments, participants gave written informed consent after the experimental procedures were explained to them. All reported normal hearing and no history of neurological disorders. The experiment was conducted in accordance with the Declaration of Helsinki and the protocol was approved by the United Ethical Review Committee for Research in Psychology (Hungary).

### Stimuli and procedures

The three experiments were similar in all aspects, except for the type of response-device and the corresponding action, which were pinching a plastic sheet, pressing a button, or tapping on a table. During the experiments, participants were sitting in an armchair, in a sound attenuated room. In all three experiments, they were instructed to perform the given action with an even tempo with a between-action interval of 3 sec. They were also instructed to remove watches and not to make overt actions to help with the timing. The experimenter demonstrated the action, but no practice trials were run.

Experiments were divided into two conditions/blocks. In the *motor-auditory* condition, individual actions elicited a 100 ms long (including linear, 5 ms rise-, and 5 ms fall times), 1000 Hz sinusoid tone delivered through headphones (HD-600, Sennheiser, Wedemark, Germany) with an intensity of 68 dB (sound pressure level, measured by an artificial head, HSUIII.2, Head Acoustics, Germany). Due to hardware constraints, there was a delay of 6 ms before tone playback started. In the *motor* condition actions did not result in a tone (and headphones were not put on). The order of the blocks was counterbalanced between participants. Both blocks consisted of 100 actions. That is, one block was approximately 5 minutes long and an experiment lasted approximately 10 minutes in total, depending on between-action interval timing.

In the Pinch Experiment a Force Sensitive Resistor (FSR; FSR Model 400, Interlink Electronics, Westlake Village, CA, USA; 0.3 mm thick, circular active area with a 5.08 mm diameter), was mounted on a thin plastic sheet. The sheet (and the FSR) was held (pinched) between the thumb and the index or middle finger of the dominant hand, with the thumb holding the sheet from above. Participants were instructed to forcefully pinch (i.e., apply a force impulse on) the sheet for a brief moment as an action. The FSR voltage signal was recorded with a voltage-divider setup using 5 V input voltage and a 10 kOhm resistor. The applied force - FSR signal relationship in this setup is well approximated by a log-linear function^22^, therefore, force was calculated from the FSR signal by an exponential transformation. The FSR signal was recorded in all experiments by using the high level input of a SynAmps2 amplifier (Compumedics Neuroscan, Victoria, Australia) with a sampling rate of 1000 Hz, with online low-pass filtering of 200 Hz (i.e., DC-200 Hz). The interaction was successful if the FSR signal exceeded 1.22 V (0.16 N), after being continuously under the threshold for at least 10 ms.

In the Button Experiment the same type of FSR was used as in the Pinch Experiment, but the FSR was mounted on a button (ST 1034, Radiohm, Tunis, Tunisia; travel distance of 0.6 ± 0.1 mm; operating force of 0.8–2.5 N). The FSR was used only to record the force signal; a successful interaction was recorded when the button was actuated.

In the Tap Experiment, an FSR with a larger active area (FSR Model 406, Interlink Electronics, Westlake Village, CA, USA, 0.46 mm thick, active area of 39.6 mm^2^) was laid out, and taped on the top of a desk. The participants’ task was to tap on the active area of the FSR with the index or middle finger (using the same finger during the whole experiment, selected by the participant before the experiment) of their dominant hand. An action was considered successful if the voltage change resulting from the applied pressure exceeded a preset threshold of 0.24 V (0.08 N), after being continuously under the threshold for at least 10 ms.

### Data processing and analysis

The first four actions in each (blocked) condition were excluded from the analyses. In the precursor study^15^, it was found that small fluctuations in the FSR signal may cause the erroneous registration of an action at the falling slope of the FSR signal (as the signal dropped below and then – due to the fluctuation - exceeded the threshold again). To exclude such potentially erroneously registered actions from the analyses, actions registered within 1 s of another action were also omitted from the analyses. For each participant, for the remaining actions the mean between-action interval within each condition was calculated and submitted to Student’s paired *t* tests in all three experiments.

To characterize force application for each successful action, a 1300 ms long epoch was extracted from the continuous FSR signal, including a 500 ms interval preceding the action. In each experiment, the applied force for each action was characterized by the maximal local peak in the corresponding FSR signal epoch. After the visual inspection of the individual FSR signals, a peak search interval was selected to accommodate most of the individual variation in peak latency. The interval was set to 0–800 ms in the Pinch; and 20–800 ms in the Press Experiment (the lower bound was 20 ms in order to avoid selecting the transient corresponding to the button-displacement, see the Results section above). In the Tap Experiment, two FSR-signal peaks were observable. To accommodate both of these peaks for all participants, two adjacent peak search intervals were used. The timepoint separating the two search intervals was individually determined between 12 and 25 ms. The upper boundary of the second interval was 800 ms. That is, for example, the two search intervals could be 0–12 ms, and 13–800 ms. The same intervals were used in both conditions. Epochs without any peaks in the given intervals were discarded from the force analyses. The ratios of epochs with an identifiable peak were compared between conditions by Students’ two-tailed, paired t-tests in each experiment. Statistical calculations were performed in R (version 3.2.3)^23^, supplementary figures were created using the ggplot2 library^24^.

To characterize the applied force for each participant, the medians of the FSR amplitudes were calculated in each condition. These were transformed to force by an exponential transformation. In the precursor study^15^ (sample size 16), force-equivalent values were compared by a two-tailed paired Student’s t-test, but due to the potential violation of the normality assumption, in the present study these were submitted to two-tailed Wilcoxon signed rank tests in each experiment. The Wilcoxon signed rank test of the data of the precursor study^15^ (with participants characterized by peak force medians in each condition) yields *T* = 0, p < 0.001, which translates to an *r* effect size^25^ of 1.000 (matched-pairs rank biserial correlation coefficient^26,27^). Based on this, at 5% alpha level, a sample size of 5 is sufficient to reach a statistical power of 80%. The sample sizes of the present study (16 in each experiment) would allow for the detection of effect sizes higher than 0.640 (corresponding to Ts lower than 25) with 80% power. Force peak latencies were averaged for each participant in each condition, and submitted to Student’s paired *t* tests for each peak. In the Tap Experiment, the two peaks were handled separately.

### Data availability

The datasets generated and analyzed during the current study are available from the corresponding author on reasonable request.

## Electronic supplementary material

Supplementary figures
