# Supplementary material for: Action-effect related motor adaptation in interactions with everyday devices
Source: Sci Rep. 2018 Apr 26;8:6592. doi: 10.1038/s41598-018-25161-w (PMC5920059; doi:10.1038/s41598-018-25161-w)
Supplement: Supplementary file 1 — Supplementary figures [file 41598_2018_25161_MOESM1_ESM.pdf]

Supplementary figures for

Action-effect related motor adaptation in interactions with everyday devices

János Horváth, Botond Bíró, Bence Neszmélyi

Captions

Figure S1. Peak forces and their latencies for each participant in the motor-auditory and motor conditions of the Pinch Experiment.

Figure S2. Peak forces and their latencies for each participant in the motor-auditory and motor conditions of the Button Experiment.

Figure S3. Peak forces and their latencies for the first force peaks for each participant in the motor-auditory and motor conditions of the Tap Experiment. The apparent discretization of the latencies is due to the sampling interval of 1 ms.

Figure S4. Peak forces and their latencies for the second force peaks for each participant in the motor-auditory and motor conditions of the Tap Experiment.

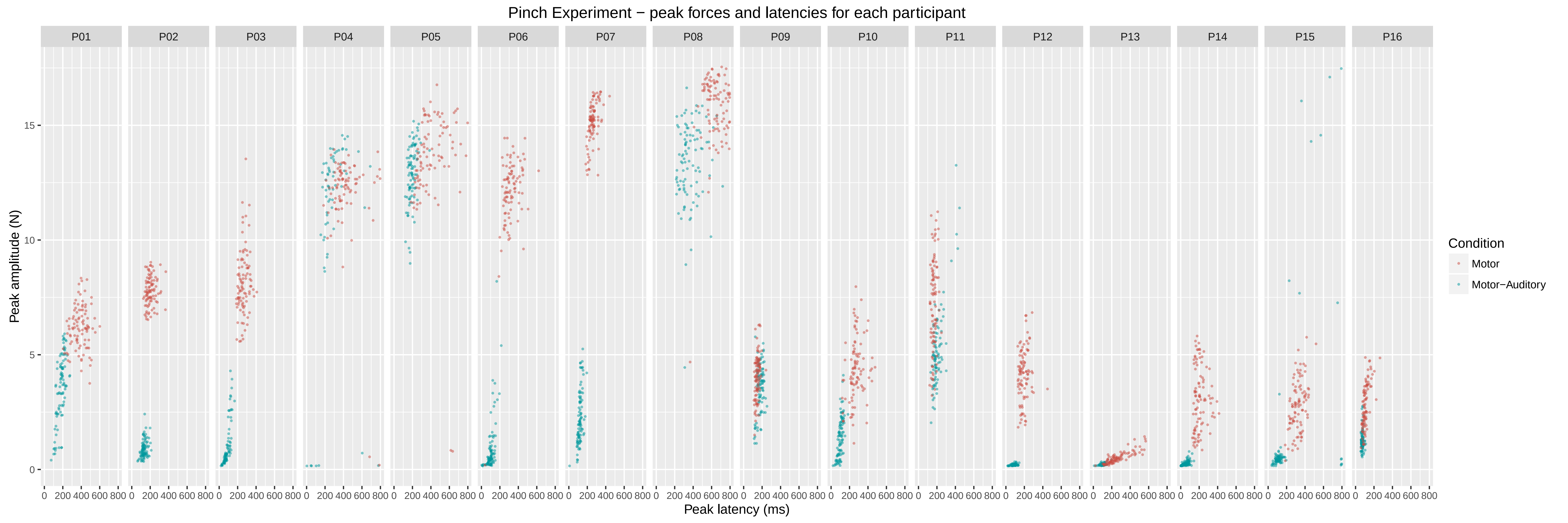

Pinch Experiment − peak forces and latencies for each participant
P01
P02
P03
P04
P05
P06
P07
P08
P09
P10
P11
P12
P13
P14
P15
P16
Peak amplitude (N)
Peak latency (ms)
0 200 400 600 800
0
5
10
15
Condition
Motor
Motor−Auditory

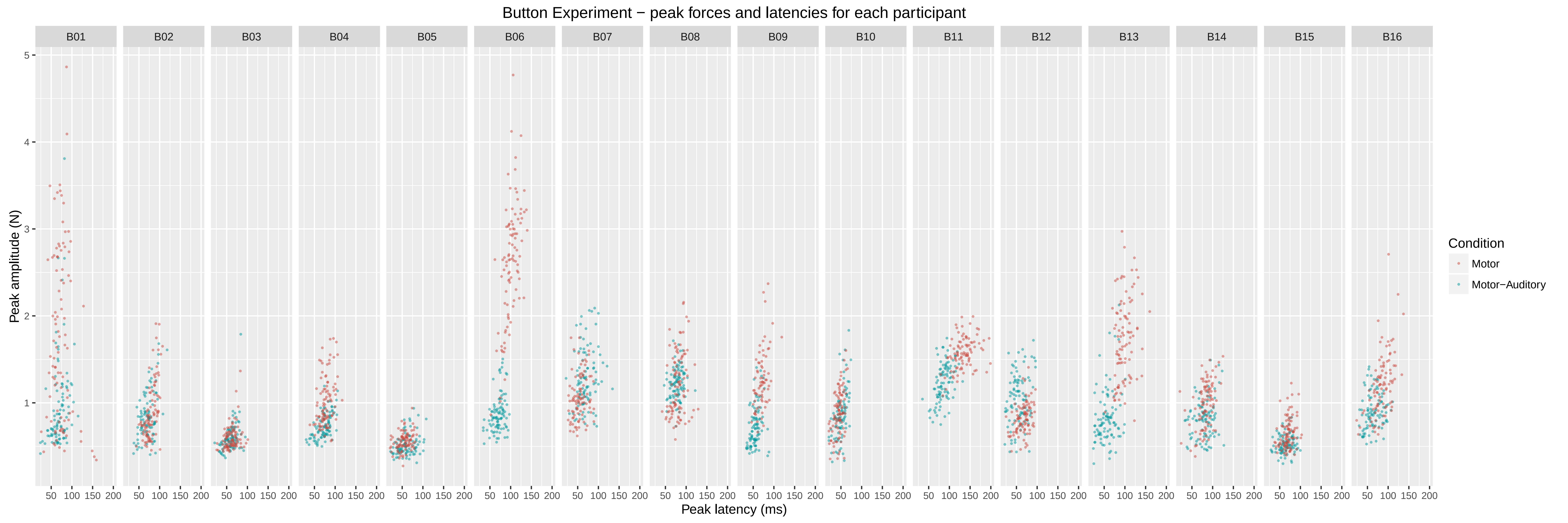
Button Experiment − peak forces and latencies for each participant
B01
B02
B03
B04
B05
B06
B07
B08
B09
B10
B11
B12
B13
B14
B15
B16
Peak amplitude (N)
Peak latency (ms)
1
2
3
4
5
50 100 150 200
Condition
Motor
Motor−Auditory

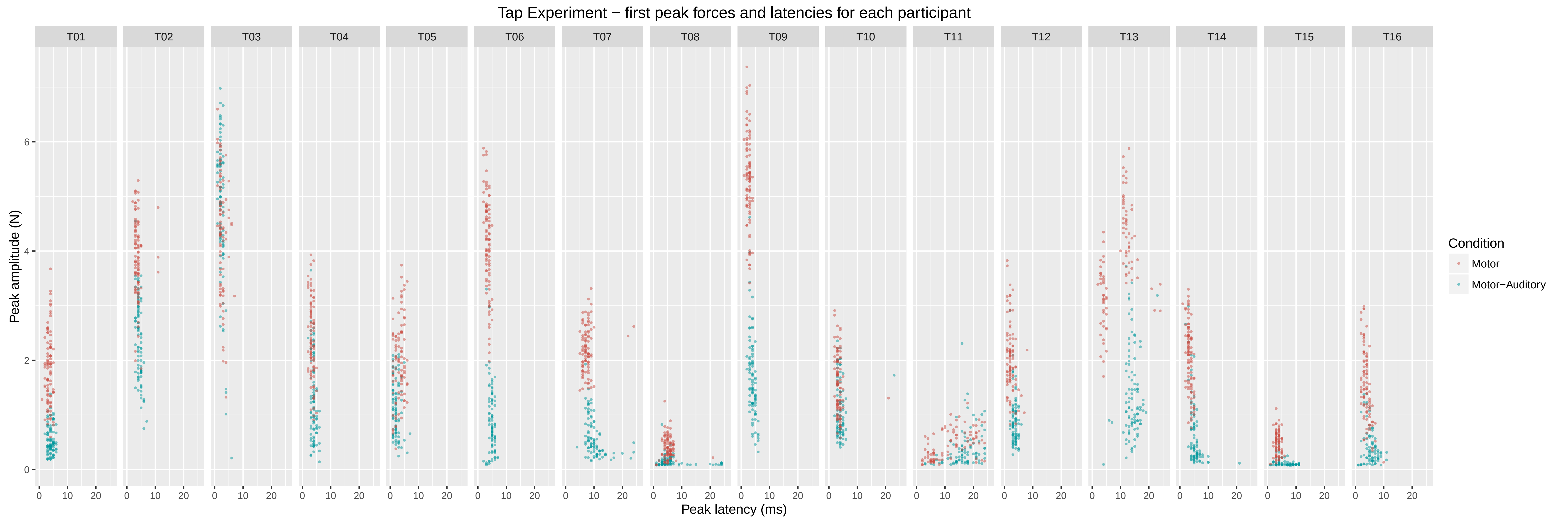

Tap Experiment − first peak forces and latencies for each participant
T01
T02
T03
T04
T05
T06
T07
T08
T09
T10
T11
T12
T13
T14
T15
T16
Peak amplitude (N)
0
2
4
6
0
10
20
Peak latency (ms)
Condition
Motor
Motor−Auditory

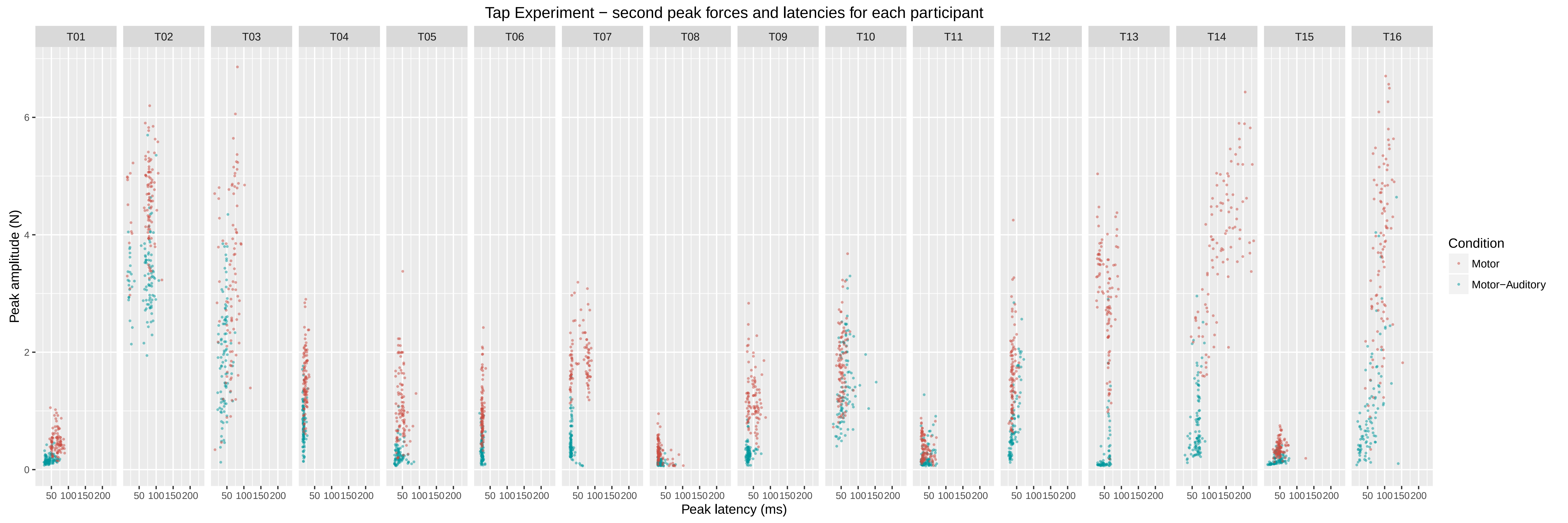
Tap Experiment − second peak forces and latencies for each participant
T01
T02
T03
T04
T05
T06
T07
T08
T09
T10
T11
T12
T13
T14
T15
T16
Peak amplitude (N)
0
2
4
6
50 100 150 200
Peak latency (ms)
Condition
Motor
Motor−Auditory
